# The Role of *Candida albicans* Virulence Factors in the Formation of Multispecies Biofilms With Bacterial Periodontal Pathogens

**DOI:** 10.3389/fcimb.2021.765942

**Published:** 2022-01-05

**Authors:** Dorota Satala, Miriam Gonzalez-Gonzalez, Magdalena Smolarz, Magdalena Surowiec, Kamila Kulig, Ewelina Wronowska, Marcin Zawrotniak, Andrzej Kozik, Maria Rapala-Kozik, Justyna Karkowska-Kuleta

**Affiliations:** ^1^ Department of Comparative Biochemistry and Bioanalytics, Faculty of Biochemistry, Biophysics and Biotechnology, Jagiellonian University in Krakow, Krakow, Poland; ^2^ Institute of Zoology and Biomedical Research, Faculty of Biology, Jagiellonian University in Krakow, Krakow, Poland; ^3^ Department of Analytical Biochemistry, Faculty of Biochemistry, Biophysics and Biotechnology, Jagiellonian University in Krakow, Krakow, Poland

**Keywords:** periodontitis, multispecies biofilms, candidal virulence factors, adhesins, moonlighting proteins, secreted aspartic proteases, quorum sensing

## Abstract

Periodontal disease depends on the presence of different microorganisms in the oral cavity that during the colonization of periodontal tissues form a multispecies biofilm community, thus allowing them to survive under adverse conditions or facilitate further colonization of host tissues. Not only numerous bacterial species participate in the development of biofilm complex structure but also fungi, especially *Candida albicans*, that often commensally inhabits the oral cavity. *C. albicans* employs an extensive armory of various virulence factors supporting its coexistence with bacteria resulting in successful host colonization and propagation of infection. In this article, we highlight various aspects of individual fungal virulence factors that may facilitate the collaboration with the associated bacterial representatives of the early colonizers of the oral cavity, the bridging species, and the late colonizers directly involved in the development of periodontitis, including the “red complex” species. In particular, we discuss the involvement of candidal cell surface proteins—typical fungal adhesins as well as originally cytosolic “moonlighting” proteins that perform a new function on the cell surface and are also present within the biofilm structures. Another group of virulence factors considered includes secreted aspartic proteases (Sap) and other secreted hydrolytic enzymes. The specific structure of the candidal cell wall, dynamically changing during morphological transitions of the fungus that favor the biofilm formation, is equally important and discussed. The non-protein biofilm-composing factors also show dynamic variability upon the contact with bacteria, and their biosynthesis processes could be involved in the stability of mixed biofilms. Biofilm-associated changes in the microbe communication system using different quorum sensing molecules of both fungal and bacterial cells are also emphasized in this review. All discussed virulence factors involved in the formation of mixed biofilm pose new challenges and influence the successful design of new diagnostic methods and the application of appropriate therapies in periodontal diseases.

## Bacteria Involved in the Periodontal Disease

Periodontal diseases, belonging to the most common oral diseases worldwide, exert far-reaching consequences for human health, being associated with further systemic diseases such as cardiovascular diseases, diabetes, insulin resistance, gastrointestinal and colorectal cancer, respiratory tract infection, Alzheimer’s disease, and adverse pregnancy outcomes (Kassebaum et al., 2014; Whitmore and Lamont, 2014; Hajishengallis, 2015; Sonti and Fleury, 2015; Vamos et al., 2015; Bui et al., 2019; Liccardo et al., 2019; Dominy et al., 2019; Liu et al., 2021). The etiology of periodontal diseases is based on the formation of the polymicrobial community residing in the subgingival compartment where further periodontal tissue colonization depends on the pathogenic potential shaped by synergistic interactions within the community or nososymbiocity (Hajishengallis and Lamont, 2012; Hajishengallis and Lamont, 2016). The mutual microbial coexistence, often based on the metabolic co-adaptations, can lead to microbe functional specialization and changes of community participant properties from commensal to pathogenic (Wright et al., 2013; Lamont and Hajishengallis, 2015).

The simplest classification of bacteria involved in periodontal disease development identifies the early colonizers, adhering to mucosal and saliva-coated tissues that include primarily Gram-positive facultative anaerobes such as *Streptococcus* spp. (*S. gordonii*, *S. mitis*, *S. oralis*, and *S. sanguinis*) and *Actinomyces* spp. (Socransky et al., 1998). They influence the local environment and collaborate with the secondary colonizers such as *Fusobacterium nucleatum*, which play the bridging function for co-aggregation, and a further adhesion of the late colonizers including *Porphyromonas gingivalis*, *Tannerella forsythia*, and *Treponema denticola*, forming the “red complex”. These species are thought to be the major etiologic agents of periodontal diseases (Suzuki et al., 2013). Such microbial succession is mediated not only by induction of changes in the local habitat, including pH and redox potential changes, or an oxygen level decrease, which favor the next colonizer existence but also include the tight intercellular interactions engaging the microbial surface adhesins (Kolenbrander et al., 2006). However, the model of successive colonization has evolved since the development of microarray techniques, showing that the infection progress is a much more complex process.

It was proposed that *P. gingivalis* plays the function of the keystone pathogen which, even at a low level of host colonization, can orchestrate the inflammation by remodeling of microbiota from benign into a dysbiotic one (Hajishengallis and Lamont, 2016). The physical interaction and diffusion of soluble factors can modulate virulence gene expression and nososymbiocity of microbes (Frias-Lopez and Duran-Pinedo, 2012). Mostly synergistic, the interactions include providing a substratum for attachment—as was found for *S. gordonii* and *P. gingivalis* dual-species biofilm (Kuboniwa et al., 2006), nutritional cross-feeding, identified for *S. gordonii* metabolic by-product (L-lactate) promoting the pathogenicity of *A. actinomycetemcomitans* (Ramsey et al., 2011), and coordinated metabolic cross-talk found for *P. gingivalis* and *T. denticola*, where the production of isobutyric acid by *P. gingivalis* stimulates *T. denticola* growth, and secretion of *T. denticola* succinate affects *P. gingivalis* cell development (see the review of Miller et al., 2019). Such manipulation can increase the pathogenicity of the whole microbial community (Hajishengallis and Lamont, 2012; Hajishengallis et al., 2012).

Moreover, the condition of the host immune system modulated by accompanying disorders or medical treatment can strongly influence polymicrobial dysbiosis and the subsequent disease progression itself (Hajishengallis and Lamont, 2021). As a keystone pathogen (Hajishengallis, 2014), *P. gingivalis* can act on the host immune system altering the Toll-like receptor response and facilitating the survival of the entire microbial community (Darveau, 2010). *P. gingivalis* also influences interleukin-8 production by gingival epithelial cells, delaying neutrophil recruitment to the infection site (Darveau et al., 1998; Hasegawa et al., 2008; Hajishengallis and Lamont, 2014). Finally, the surface exposed or secreted cysteine proteinases (gingipains), which activate or degrade complement factors C3 and C5, may lead to the avoidance of complement-mediated detection of accompanying microbiota (Hajishengallis and Lamont, 2012; Hussain et al., 2015).

These findings have also indicated that the analysis of mixed-species community formation should be extended to the possible inter-kingdom interactions between bacterial and commensal fungal species belonging to the *Candida* genus, especially *Candida albicans* which were found to influence the colonization or metabolic activity of early, bridging, and keystone pathogens of periodontal disease, leading to the onset of severe caries *in vivo* (Wu et al., 2015; Hwang et al., 2017; Kim et al., 2017; Sztukowska et al., 2018; Bartnicka et al., 2019; Bartnicka et al., 2020).

## *C. albicans* Ability to Form a Multispecies Biofilm Community in Periodontal Diseases

**Figure 1:**
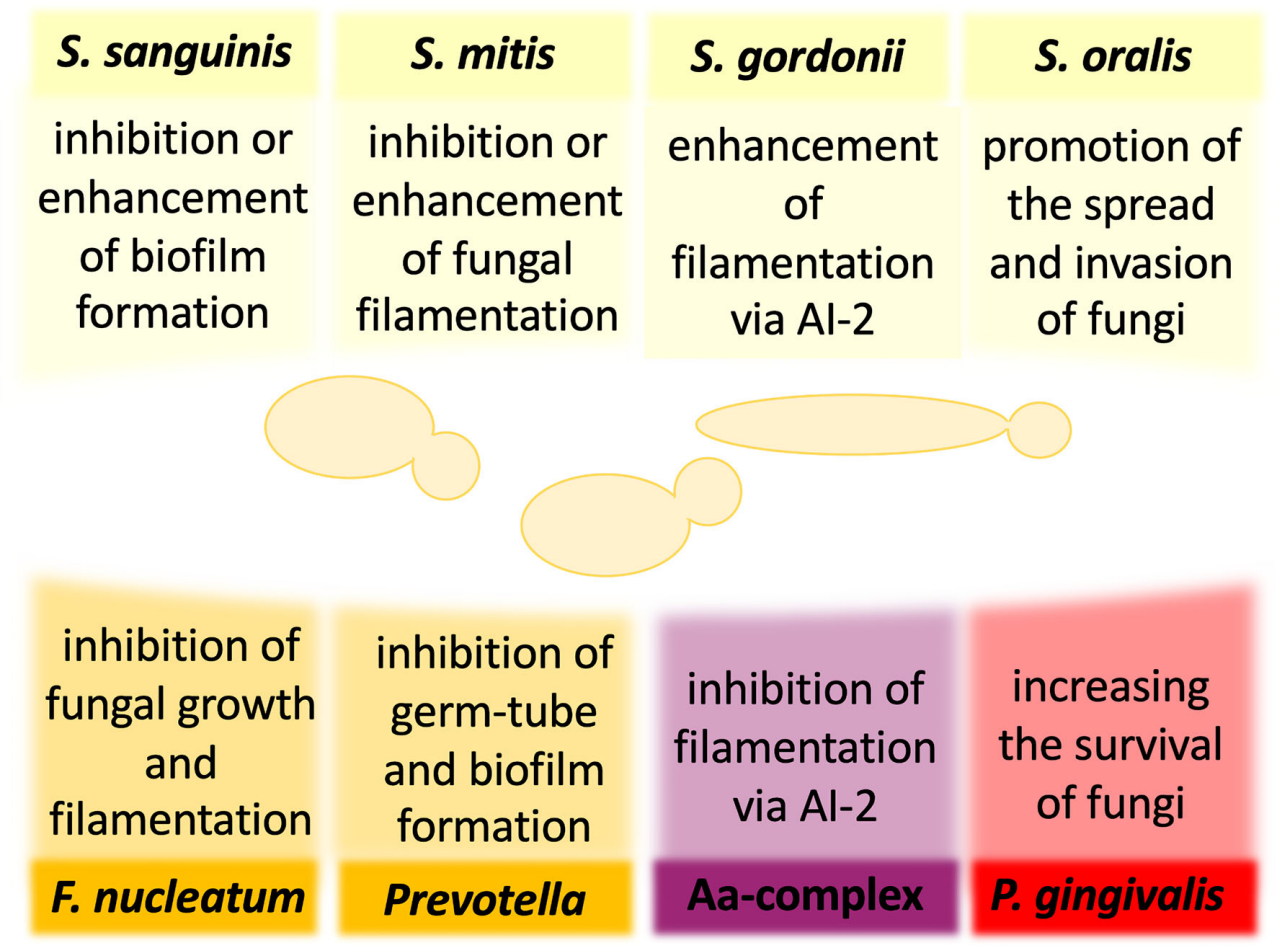
The influence of oral bacteria on the virulence and pathogenicity of *Candida albicans*. The interactions of *C. albicans* and oral bacteria occur through many mechanisms and may result in either reduction or enhancement of fungal virulence. For some bacteria, the published preliminary analyses have not yet determined unequivocally the nature of the influence of bacteria on the development of mixed infection with *C. albicans.* AI-2, autoinducer 2; Aa, *Aggregatibacter actinomycetemcomitans*.

*C. albicans* is the most commonly identified yeast in the oral cavity of healthy people (Ghannoum et al., 2010; Baumgardner, 2019). A preliminary hypothesis that this fungus may be involved in the development of chronic periodontal disease was based on the analysis of samples taken from supragingival and subgingival sites of patients with chronic periodontitis for whom a higher *C. albicans* colonization rate was shown compared with healthy individuals (Urzúa et al., 2008). Moreover, the recent finding that *C. albicans* is the keystone commensal in the oral cavity, which may form interspecies networks with different bacteria, stressed the importance of this fungus in periodontitis (Diaz et al., 2014; Janus et al., 2016; De-La-Torre et al., 2018; Krüger et al., 2019; Young et al., 2020; Jabri et al., 2021). Changes in the biofilm bacterial composition in ecological niche shared with fungi may encourage interspecies cooperation for the benefit of all interacting partners, like evading host immune system or enhancing biofilm properties, but may also become an opportunity to compete for available space or nutrients (**Figure 1**). Unfortunately, the biological consequences of the interspecies interactions within biofilm in the course of periodontitis, for both the particular microorganisms and the host, still largely remain unclear.

For the group of early colonizers, including *S. sanguinis*, *S. oralis*, *S. mitis*, and *S. gordonii*, the outcome of interactions with fungi is not sufficiently well understood, and the available reports often contradict to each other. However, all these species were shown to co-aggregate with clinical isolates of *C. albicans* (Jenkinson et al., 1990). In the case of *S. sanguinis*, one of the first reports suggested that the type of interspecies interactions depends on the conditions of the specific niche, from which the bacteria were derived. Prior exposure of *C. albicans* oral isolates to *S. sanguinis* isolated from healthy and HIV-infected individuals resulted in an inhibition or promotion of candidal germ tube formation, respectively (Nair et al., 2001). Further *in vitro* analyses showed that the addition of *S. sanguinis* and *S. mitis* cells to *C. albicans* ATCC 18804 cells pre-incubated to promote initial fungal adhesion considerably reduced fungal CFU in mixed biofilm compared with single-species fungal biofilm, while also the presence of *S. mitis*, but not *S. sanguinis*, reduced *C. albicans* filamentation (do Rosário Palma et al., 2019). These data contrast with a study of biofilm formation on the salivary flow which showed that *C. albicans* strain ATCC SC5314 exhibits an enhanced biofilm-formation capacity in the presence of *S. sanguinis* and *S. gordonii* (Diaz et al., 2012). Hence, the question remains whether or not a group of early colonizers modulates fungal biofilm formation to influence oral health or disease progression in the host organism. Furthermore, the study using *Galleria mellonella* as an alternative model of mixed bacterial–fungal infection did not show any significant changes in fungal abundance and morphological changes during 12 h of co-infections of larvae by *C. albicans* strain ATCC 18804, *S. mitis*, and *S. sanguinis* (do Rosário Palma et al., 2019). However, for other bacterium from the group of early colonizers—*S. gordonii*—a promotion of *C. albicans* strain ATCC SC5314 biofilm formation was demonstrated (Bamford et al., 2009). Since the *S. gordonii* luxS mutant deficient in the production of quorum-sensing molecule (QSM)—autoinducer 2 (AI-2)—possessed a reduced ability to induce *C. albicans* hyphal formation and significantly reduced biomass of mixed biofilm, it could be suggested that fungal–bacterial interactions involve chemical signals that influence the development of interspecies communities (Bamford et al., 2009). Similar results were observed for *S. oralis* in the mucosal tissue model, showing also that *C. albicans* strain ATCC SC5314 promoted bacterial biofilm growth, consequently increasing further fungal invasion on the oral mucosa (Diaz et al., 2012). The use of an *in vitro* oral infection model showed that *S. oralis* promotes the spread of *C. albicans* strain ATCC SC5314 and the development of systemic infection. Mice infected with both pathogens presented an excessive inflammatory response, dependent on TLR2 signaling, and increased neutrophilic activity (Xu et al., 2014b). Thus, while *S. oralis* cells do not have significant virulence properties, they provide suitable conditions for promoting the virulence of *C. albicans*.

One of the possible advantages of the fungal interaction with *S. gordonii* and *S. sanguinis* is an opportunity to utilize bacterial cells as a kind of scaffold, as bacteria by their binding to enamel prevent *C. albicans* removal from the oral cavity and initiate the formation of fungal biofilm that may be additionally stabilized by the extracellular release of bacterial DNA (O’Sullivan et al., 2000; Bamford et al., 2009; Xu et al., 2014a; Jack et al., 2015). In *S. sanguinis*, three pilus proteins PilA, PilB, and PilC were identified as receptors through which bacteria bind to components of saliva and become immobilized on the tooth surface (Okahashi et al., 2011). Moreover, it has been documented that the *C. albicans* interaction with *S. gordonii* is mediated by streptococcal cell wall-anchored proteins SspA and SspB with an additional involvement of proline-rich proteins from saliva adsorbed on the surface of bacteria and recognized by *C. albicans* cells (O’Sullivan et al., 2000; Bamford et al., 2009; Xu et al., 2014a).

For the bridging colonizer of the oral cavity—*F. nucleatum*—one of the first reports suggested that the co-aggregation with clinical isolates of *C. albicans* was dependent on the temperature of fungal culture (Jabra-Rizk et al., 1999), whereas other studies showed that co-agglutination was hindered after heat treatment and trypsinization of *F. nucleatum* cells, suggesting the important role of bacterial surface proteins in these interactions (Bagg and Silverwood, 1986; Grimaudo and Nesbitt, 1997). Extensive *in vitro* studies have shown that the physical contact between *F. nucleatum* and *C. albicans* strain SN152, which is isogenic to strain ATCC SC5314, results in inhibition of fungal growth and filamentation without altering fungal cell viability (Bor et al., 2016). As *C. albicans* blastospores were demonstrated to be less susceptible to attack by RAW 264.7 macrophages than the filamentous cells, it was postulated that arresting the fungal morphological changes by bacteria weakens the host immune response, for the benefits for both partners, allowing them to go unnoticed and spread further to other organs (Bor et al., 2016). This hypothesis was supported by the experiments in which the co-incubation of *F. nucleatum* and *C. albicans* significantly reduced the production of monocyte chemoattractant protein-1 (MCP-1) and tumor necrosis factor (TNF-α) compared with the response of the host cells during single-species infection (Bor et al., 2016).

Furthermore, for *P. nigrescens*, one of the representatives of the genus *Prevotella* classified as the bridging colonizer in the oral cavity, it was shown that 48-h coculture with *C. albicans* oral isolates inhibited the formation of fungal biofilm on the surface of polystyrene plastic, manifested by the reduction of viable biofilm cell mass (Thein et al., 2006). In the presence of a large number of *P. nigrescens* cells (10^7^/ml), a significant decrease in the viability of *C. albicans* was also noticed, suggesting that the modulation of fungal biofilm formation correlated with the number of bacterial cells (Thein et al., 2006). Other studies showed an inhibitory effect of *P. intermedia*, isolated from the subgingival plaque of HIV-infected patients, on the formation of *C. albicans* germ tubes (Nair et al., 2001). A similar effect was also observed for another bacterium from the group of bridging colonizers—*Campylobacter*—that through the secretion of bacteriocin-like substances with antimicrobial activity inhibited the growth of *C. albicans* laboratory strain ATCC 44859 as detected using measurements of the zone of inhibition (Workman et al., 2007).

The presence of the highly pathogenic bacterial periodontal pathogen belonging to the red complex—*P. gingivalis*—not only induced germ-tube formation by both oral isolates and *C. albicans* reference strain ATCC 10231, resulting in the generation of a more invasive fungal phenotype, but also stimulated the adhesion and growth of fungal hyphae on the artificial surface (Nair et al., 2001; Bartnicka et al., 2019). However, other studies showed that the presence of many bacteria (10^7^/ml) was positively correlated with a reduction in fungal oral isolates viability, indicating that they can inhibit the formation of *C. albicans* biofilm (Thein et al., 2006). Rather than to the blastospores or pseudohyphal form, *P. gingivalis* easily adhered to the hyphae of *C. albicans*, and the interspecies adhesion was mediated by bacterial InlJ—a homolog of the internalin protein family—and by gingipain RgpA (Sztukowska et al., 2018; Bartnicka et al., 2019). Recent extensive *in vivo* and *in vitro* studies using *C. albicans* reference strain ATCC 10231 have determined the effect of a dual-species infection on the host (Karkowska-Kuleta et al., 2018; Bartnicka et al., 2020). Using monocyte-like cell line THP-1, it was shown that the coexistence of bacteria with *C. albicans* downregulated the expression of genes, encoding MCP-1, TNF-α, and interleukin-1β (IL-1β), compared with the response generated by the host cells in contact with monospecies bacterial infection (Karkowska-Kuleta et al., 2018). Suppressing the host immune response during dual-species infection was also postulated from the observed lowered neutrophil response, manifested by a significantly reduced elastase activity than in the case of pure bacterial biofilm (Bartnicka et al., 2020). A well-established model used to *in vivo* mimic the dynamics of the infection process is the subcutaneous chamber mouse model in which titanium or stainless steel wire coils are implanted subcutaneously in the dorsolumbar region of each mouse. The possible access to the contents of the chamber after the healing period allows it to be used as a biological compartment for studying host–microbe interactions (Genco et al., 1991; Houri-Haddad et al., 2000). Recent study by Bartnicka et al. (2020) based on this model indicated reduced bacterial CFUs in the first days of mixed infection, while the presence of *P. gingivalis* increased the proliferation of *C. albicans* on a longer time scale. Analysis of the microbial burden in the organs isolated from infected mice confirmed the reduced bacterial load in the dual-species infection compared with bacterial infection (Bartnicka et al., 2020). Accordingly, it was shown that *P. gingivalis* colonization of a host previously infected with *C. albicans* caused milder inflammation, leading to prolonged survival of the infected mice, and confirming the chronic nature of the dual-species infection (Bartnicka et al., 2020).

For another microorganism highly associated with aggressive periodontal disease, such as *Aggregatibacter actinomycetemcomitans*, a Gram-negative bacterium belonging to the Aa complex, the ability to inhibit *C. albicans* filamentation and biofilm formation, mediated by secretory AI-2, was previously demonstrated (Bachtiar et al., 2014; Baker et al., 2017). Taking into account the fact that *C. albicans* blastospores are less sensitive to the action of host macrophages (Bor et al., 2016), it can be concluded that the bacteria, by suppressing fungal biofilm formation, indirectly protect *C. albicans* from the host immune system. The recent studies of polymicrobial biofilms by Bhardwaj et al. (2020) showed that the simultaneous presence of *A. actinomycetemcomitans* and Gram-positive bacteria—*S. gordonii* and *S. mutans*—significantly accelerated the growth of *C. albicans* reference strain ATCC 24433. Moreover, an increased ability of polymicrobial communities to induce an inflammatory response of host cells was demonstrated. Both biofilms and biofilm supernatants significantly induced the increase of TNF-α and IL-8 at the gene and protein levels (Bhardwaj et al., 2020). In a recent study by Young et al. (2020), two biofilm models were used to investigate the role of *C. albicans* in the multispecies community. The first model of hard tissue related to caries initially involved a 24-h co-incubation of two pioneering species, *C. albicans* laboratory strain 3153A and *S. mutans* (10^7^ CFU/ml of bacteria and fungi in an equal volume), followed by the addition of a mixture of four species—*F. nucleatum*, *A*. *naeslundii*, *Veillonella dispar*, and *Lactobacillus casei* (10^7^ CFU/ml for each bacterium), which were co-incubated for the next 4 days to form a biofilm structure. Whereas the second model of soft-tissue related to periodontitis/denture stomatitis contained ten bacteria species—*S. oralis*, *S. mitis*, *S. intermedius*, and *F. nucleatum*—which were pioneering biofilm species, but also *F. nucleatum* ssp. *vincentii*, *A. naeslundii*. *V. dispar*, *P. gingivalis*, *P. intermedia*, and *A. actinomycetemcomitans*. It has been shown that the addition of *C. albicans* increases the mass and rate of biofilm metabolism in both tested systems, causing a simultaneous quantitative change in the bacterial composition (Young et al., 2020). Although *V. dispar* and streptococci remained the main species, there was an increase in *P. gingivalis* and *F. nucleatum* presence in the soft-tissue and hard-tissue biofilm, respectively. In both systems, the presence of *C. albicans* significantly raised the pH of the supernatant to more neutral, which was associated with a decrease in the lactate level. In addition, an increase in the activity of superoxide dismutase (SOD) in the biofilm of soft tissues was noted and according to the study’s authors, this result was associated with an increased presence of anaerobic bacteria (Young et al., 2020).

## Fungal Virulence Factors Important for Biofilm Community Formation

The armory of virulence-related molecules that *Candida* fungi have at their disposal includes surface-located adhesins and invasins, atypical cell wall proteins (moonlighting proteins), cell wall polysaccharides—mannans, glucans, and chitin, secreted hydrolytic enzymes, toxins, and low molecular weight compounds like quorum sensing molecules and other secondary metabolites (for comprehensive reviews see Hoyer and Cota, 2016; Snarr et al., 2017; Rapala-Kozik et al., 2018; Rodrigues and Černáková 2020; Satala et al., 2020a). These factors are involved in interactions with host proteins and cells, the evasion of host immune system, and intra- and interspecies communication. Moreover, such mechanisms related to virulence like hyphae formation, biofilm production, phenotypic switching, and enhanced stress tolerance are highly responsible for the effectiveness of fungi as pathogens (Staniszewska, 2020). All of these virulence factors and mechanisms might be affected by multifaceted interactions with a variety of bacterial species during co-colonization of subgingival sites and the formation of a mixed biofilm community in the course of periodontal disease. Better understanding of these interaction mechanisms could contribute in the future to taking control of microbial infections related to the formation of multispecies consortia.

**Figure 2:**
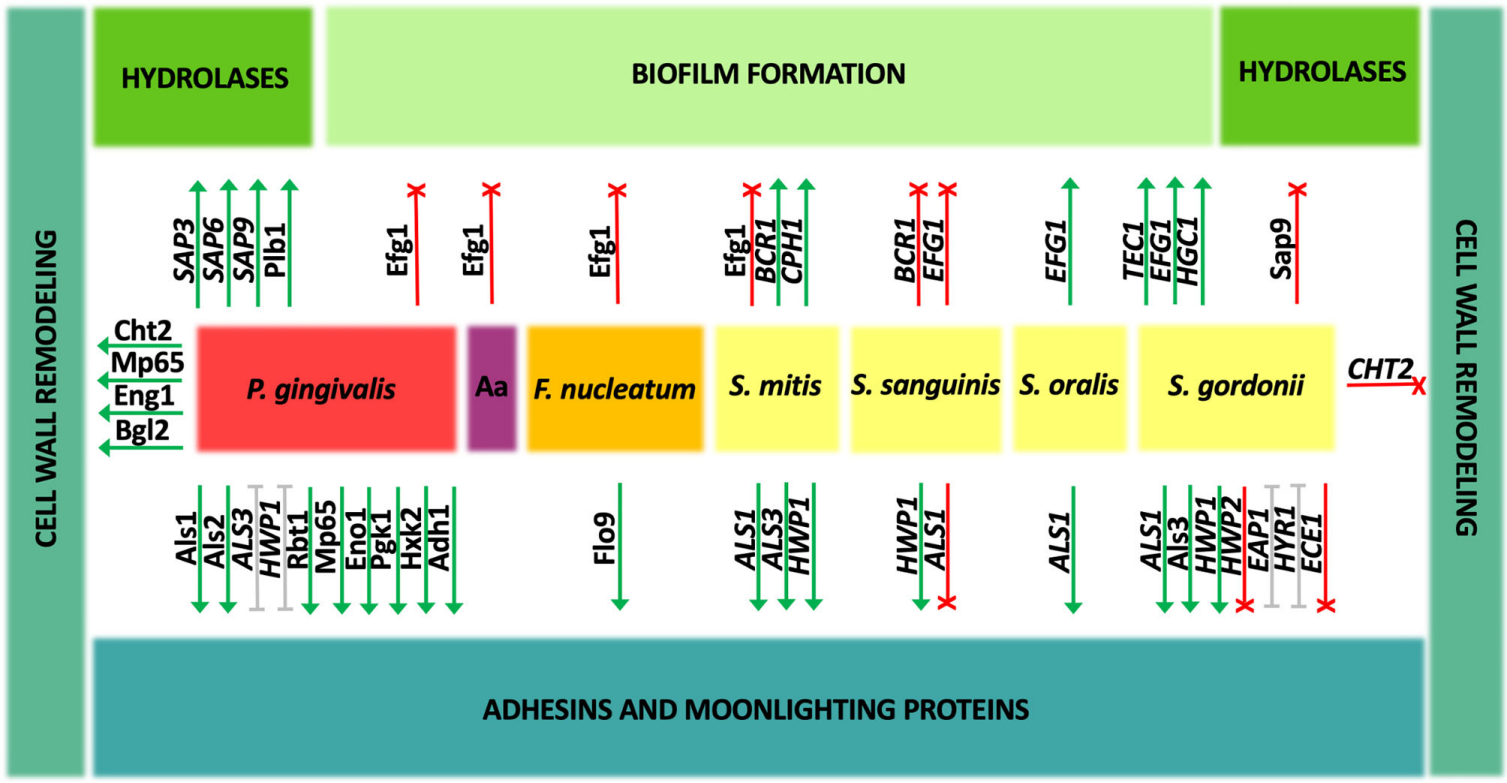
Change in the gene expression and amounts of *Candida albicans* proteins during coexistence with oral bacteria. Interspecies interactions may show either an enhancement (green) or inhibitory (red) effect on individual *C. albicans* virulence factors. For some aspects (gray), the described effect is ambiguous and strictly depends on the experimental conditions. Aa, *A. actinomycetemcomitans*.

One of the courses of research on the contribution of different fungal virulence factors in interactions with bacteria is the study of the expression of selected master regulatory genes involved in controlling such processes as fungal filamentation, adhesion, or biofilm production (**Figure 2**). Also, the expression level of particular genes encoding individual virulence factors, like adhesins, cell wall remodeling enzymes, or secreted hydrolases may be analyzed after fungal contact with bacterial companions. Another research approach is the use of *C. albicans* deletion mutant strains or yeast strains with overexpressed individual virulence factors or key transcriptomic regulators of fungal virulence-related processes for the formation of mixed biofilms or contact with bacteria. In addition, the study of changes in the fungal proteome after exposure to bacteria may also provide valuable information on mutual influences. Finally, the analyses of direct physicochemical interactions between native molecules produced by cells of various pathogens are successfully used to characterize microbial contact in a mixed community. All these approaches, although they differ significantly, also complement each other, allowing for a more comprehensive depiction of the complex relations within the subgingival plaque.

### Main Adhesins and Moonlighting Proteins

The major *C. albicans* adhesins comprise a group of typical cell wall proteins highly glycosylated and covalently bound to the cell surface, often *via* the glycosylphosphatidylinositol (GPI) anchor. Their main representatives with confirmed participation in the binding of various ligands are proteins from agglutinin-like sequence family (Als1-7, Als9), enhanced adherence to polystyrene protein (Eap1), hyphal cell wall protein 1 (Hwp1), and structurally related proteins Hwp2 and Rbt1 (Staab et al., 1999; Li and Palecek, 2003; Younes et al., 2011; Monniot et al., 2013; Hoyer and Cota, 2016).

In the group of oral streptococci, the problem of co-adhesion of bacteria and fungi during biofilm development has been quite decently studied and attempts have been made to indicate the mechanisms of these interactions. It was demonstrated that when *C. albicans* strain ATCC 18804 creates biofilm together with *S. mitis*, the expression levels of genes encoding major adhesins, agglutinin-like sequence proteins *ALS1* and *ALS3*, and hyphal cell wall protein *HWP1* were significantly upregulated, whereas in the coexistence in biofilm with *S. sanguinis*, only the gene expression for *HWP1* was upregulated, for *ALS3* the expression did not change, and for *ALS1* downregulation was observed (do Rosário Palma et al., 2019). On the other hand, the increase in *ALS1* gene expression was noticed during the formation of mature biofilm by *C. albicans* strain SC5314 and *S. oralis* at a ratio of 1:10 and the *als1*Δ/Δ deletion mutant strain was deficient in co-aggregation with this bacterial species (Xu et al., 2017). Nevertheless, so far most information on the adhesion of *C. albicans* to streptococci is available for *S. gordonii*. Studies using *C. albicans* two control strains CAI12 and DAY185 and mutant strains deprived of Als1 and Als3 proteins showed the joint participation of these two proteins in the interactions with *S. gordonii*, with little contribution from other adhesins from Als family—Als2 or Als4 (Hoyer et al., 2014). Furthermore, a co-incubation of *C. albicans* wild-type strain SC5314 and *S. gordonii* for 1 h at 37°C in YPT-Glc medium increased the expression of several genes encoding cell wall proteins equipped with GPI anchor, including adhesin-encoding genes *ALS1*, *HYR1*, and *EAP1* (Dutton et al., 2016a). Of these genes, the latter, when overexpressed on the surface of *Saccharomyces cerevisiae* cells, was indicated as responsible for direct interactions with *S. gordonii* planktonic cells (Nobbs et al., 2010). In this research, employing *S. cerevisiae* as a surrogate host for candidal adhesive proteins, also Als3 was demonstrated as a protein that binds bacteria strongly, while other adhesins, Hwp1 and Rbt1 bound streptococcal cells significantly weaker (Nobbs et al., 2010). The role of Als3 adhesin in binding to *S. gordonii* cells was also confirmed using a *C. albicans* 1843 *als3*Δ*/als3*Δ deletion mutant, for which the co-aggregation of fungal cells with preformed bacterial monolayer was nearly completely abolished, and as a bacterial partner in these interactions the *S. gordonii* protein SspB, representative of antigen I/II family polypeptide adhesins, was indicated (Silverman et al., 2010). In opposition to these results are the observations presented by Montelongo-Jauregui et al. (2019), as in their studies the *C. albicans als3Δ/Δ* mutant strain was capable to form biofilms together with *S. gordonii*, a finding explained by substantial differences in the experimental approach and the media used. In this case, the mixed biofilm was initiated by the simultaneous introduction of fungi and bacteria at a ratio of 1:10 into the well of the microplate and formed for further 24 h in basal medium mucin synthetic saliva, which was able to restore the ability to form a single biofilm by mutant strains of *C. albicans*. Moreover, in the studies carried out by Klotz et al. (2007) that exploited also *S. cerevisiae* cells carrying *C. albicans* adhesins, Als5 was indicated as a protein sufficient for co-aggregation of fungi with *S. gordonii*.

In the continuation of research carried out by Nobbs et al. (2010), specific fragments of the Als3 protein, namely, N-terminally located fragments comprising aa 166–225, 218–285, 270–305, and 277–286 were found to be primarily responsible for the interaction with *S. gordonii* cells and SspB protein, whereas the lack of amyloid-forming region (AFR) (aa 325–331) and central repeat domain (aa 434–830) reduced the binding with bacteria only by 50% (Bamford et al., 2015). In similar studies, the use of *C. albicans* strains with site-directed Als3 mutations suggested the significant contribution of the N-terminal domain-located peptide-binding pocket (PBC) in the binding of streptococcal SspB and the lack of involvement of the AFR fragment in these interactions (Hoyer et al., 2014).

Interestingly, Salvatori et al. (2020) demonstrated that heat-fixed culture supernatants from *S. gordonii* induced the formation of two phenotypically different types of microcolonies by *C. albicans*. In the prevailing type of floating dense microcolonies detached from the surface, an increase in the expression of the adhesin genes *ALS3* and *HWP1* was observed, alongside with the decrease in the expression of genes *ECE1*, *HYR1*, *EAP1*, and *HWP2*, thus prompting the authors to postulate that this is a phenotype associated with the facilitated spread of fungi in the organism on the one hand, and the maintenance of the commensal state of the fungi on the other hand (Salvatori et al., 2020).

The relative changes in the expression of genes, encoding adhesins *ALS3*, *EAP1*, and *HWP1*, comprised their upregulation during growth for 72 h in a mixed biofilm formed by *C. albicans* ATCC 90028 and four different species of oral bacteria—*S. sanguinis*, *S. gordonii*, *Actinomyces odontolyticus*, and *Actinomyces viscosus*, whereas with the additional presence of *P. gingivalis* these expression levels were significantly lower than for mixed biofilms without this strict anaerobe (Morse et al., 2019). Moreover, the formation of bacterial–fungal biofilms including *P. gingivalis* resulted in a different pattern of *C. albicans* gene expression compared with biofilms produced only by *C. albicans*, and the *ALS3* gene was downregulated under these conditions (Morse et al., 2019).

In the case of the bacterial species other than streptococci, which are related to the development of periodontitis, there are neither many reports on the direct interactions of particular molecules during mixed bacterial–fungal biofilm formation nor the involvement of individual virulence factors. In the case of *F. nucleatum*, Grimaudo and Nesbitt demonstrated in 1997 that the cell wall carbohydrate or carbohydrate-containing molecule is involved in the interactions with proteinaceous components presented at the surface of bacteria, as the addition of mannose, glucosamine, and alpha-methyl mannoside significantly reduced microbial co-aggregation. Subsequently, with the use of the *C. albicans* mutant library, the strain defective in the expression of putative adhesin-like cell wall mannoprotein Flo9 demonstrated a significant reduction in co-aggregation and biofilm formation with *F. nucleatum*, and this process was significantly disturbed in the presence of mannose. Therefore, this particular protein might be indicated as a *C. albicans* partner in direct interactions with *F. nucleatum*, and the important role of its glycosylation was also confirmed with the *C. albicans* mutant strain deprived of alpha-1,6-mannosyltransferase Och1, which was also characterized by weakened aggregation with bacteria (Wu et al., 2015).

In a study on the transcription levels of *C. albicans* genes during the formation of mixed-species biofilm with *S. mutans* in the presence of spent media from *A. actinomycetemcomitans* culture, the expression level of *ALS3* and *HWP1* genes in fungal cells was decreased probably as a result of the action of AI-2 produced by the latter species (Bachtiar and Bachtiar, 2020).

The direct contact between *C. albicans* and *P. gingivalis* during biofilm formation is based on the binding of various adhesive proteins. Initial detailed studies on the interactions between these two pathogens concerned the changes in the expression of genes encoding fungal adhesins during biofilm formation under anaerobic conditions for 72 h on the titanium surface with the concentration of microorganisms ~1 × 10^5^ CFU/ml for *C. albicans* and ~1 × 10^7^ CFU/ml for bacterial species (Cavalcanti et al., 2016). Under these conditions, the expression level of adhesin *HWP1* was decreased, and no statistically significant changes were observed for *ALS1* and *ALS3* genes. In the case of the latter gene, the significant upregulation was noticed in mixed biofilm composed additionally of *S. sanguinis* and *S. mutans*; however, this level was similar also in the biofilm composed of fungi and streptococci without *P. gingivalis*. On the other hand, the level of *HWP1* gene expression was reduced in such a multispecies biofilm compared with the biofilm formed by *S. sanguinis*, *S. mutans*, and *C. albicans* ATCC 90028, and comparable to that in a single fungal biofilm, thus proving the downregulation of this gene in the presence of *P. gingivalis* (Cavalcanti et al., 2016).

Sztukowska et al. (2018) showed that *P. gingivalis* InlJ internalin-family protein interacts with *C. albicans* SC5314 hypha-associated adhesin Als3, as the binding of *C. albicans* mutant strain *als3*Δ to *P. gingivalis* significantly decreased compared with the wild strain and the binding was additionally confirmed using *S. cerevisiae* cells overexpressing *C. albicans* Als3. Surprisingly, bacterial FimA-deficient mutants adhered to fungal hyphae comparably to the wild type, suggesting a marginal role of fimbriae in the aforementioned heterotypic pathogenic interactions (Sztukowska et al., 2018). Further analyses of direct interactions between *C. albicans* ATCC 10231 and *P. gingivalis* in mixed biofilm in the simultaneous model of interaction were presented in the work by Bartnicka et al. (2019). Under aerobic conditions, a 3-h contact during mixed biofilm formation with *P. gingivalis* wild strain resulted in the increase in the expression levels of *ALS3* and *HWP1* genes, while *ALS7* expression remained unchanged, whereas under anaerobic conditions, unfavorable for fungi, the expression of *ALS3* was reduced, and that for *ALS7* and *HWP1* remained at the same level as in single fungal biofilm. In the case of using a bacterial mutant strain ΔKΔRAB devoid of proteolytic enzymes—gingipains—the increase in gene expression for all three adhesins was evident under both conditions tested (Bartnicka et al., 2019). Subsequently, a proteomic analysis using cell-surface shaving with trypsin was performed to identify fungal proteins exposed on the cell wall during mixed biofilm formation for 24 h in the simultaneous model of interaction. Corroborating the results of gene expression, the surface exposition of the Als3 protein significantly increased in biofilm formed in normoxia with the *P. gingivalis* ΔKΔRAB strain. Moreover, under these conditions also Als1, Rbt1, and Als2 adhesins were overproduced by *C. albicans* during mixed biofilm formation, the latter protein under anoxic conditions. Additionally, the increase in the amount of cell surface mannoprotein Mp65, which possesses both adhesive properties and activity in cell-wall glucan metabolism, was demonstrated for both tested bacterial species and biofilm growth conditions. It should be taken into account that all these fungal proteins may be targeted for proteolytic processing by bacterial gingipains during direct contact between pathogens (Bartnicka et al., 2019).

Additionally, the expression of the *ENO1* gene, encoding the cytosolic glycolytic enzyme, enolase, was unaffected during 3 h of *C. albicans* contact with any bacterial cells in normoxia and only slightly decreased after contact with *P. gingivalis* ΔKΔRAB in anoxia. *C. albicans* enolase belongs to the group of moonlighting proteins—proteins performing a completely different function in a location different from the original one—and is repeatedly identified at the fungal cell surface where it is involved in the binding of different host proteins (Satala et al., 2020a; Satala et al., 2020b; Karkowska-Kuleta et al., 2021). Further proteomic studies showed the overproduction of surface-localized Eno1 during biofilm formation with *P. gingivalis* wild type and the mutant strain in both normoxia and anoxia. Among other surface-displayed moonlighting proteins, also the production of *C. albicans* phosphoglycerate kinase (Pgk1) and hexokinase 2 (Hxk2) increased under aerobic conditions upon contact in biofilm with *P. gingivalis* wild type strain, and alcohol dehydrogenase 1 (Adh1) in anoxia and gingipain-depleted mutant strain (Bartnicka et al., 2019).

Of these abovementioned fungal cell surface proteins, a selected few were purified from *C. albicans* ATCC 10231 cell walls—Als3 and Eno1—or culture supernatants—Mp65—and their ability to bind to the *P. gingivalis* cells was demonstrated. They bound to both the wild strain and the gingipain-deficient strain, indicating the presence of numerous binding partners on the bacterial surface. Finally, the direct interactions between these fungal proteins and the surface bacterial gingipain RgpA, possessing a hemagglutinin adhesive domain in addition to the catalytic one, were verified in thermodynamic and kinetic analyses with surface plasmon resonance (SPR) measurements (Potempa et al., 2003; Bartnicka et al., 2019). A higher binding affinity was indicated for the Eno1–RgpA complex than for two other proteins, being typical adhesins. This may indicate that this moonlighting protein abundant in biofilm is a significant support in bacterial–fungal interactions in addition to typical candidal adhesins. Moreover, these three fungal proteins, Als3, Mp65, and Eno1, were identified as citrullinated by the bacterial enzyme—peptidylarginine deiminase (PPAD)—during the interactions of *C. albicans* ATCC 10231 with *P. gingivalis* at a ratio of 1:10 in mixed biofilm formed for 24 h in the simultaneous model of interaction in RPMI 1640 medium. Additionally, also other surface-exposed candidal moonlighting proteins were prone to this modification, including Hxk2, Pgk1, Adh1, pyruvate decarboxylase (Pdc1), and glyceraldehyde-3-phosphate dehydrogenase (Tdh3) (Karkowska-Kuleta et al., 2018; Karkowska-Kuleta et al., 2020). This modification may influence their role in biofilm, as it was pointed out that citrullination by PPAD is important in the process of mixed biofilm formation because the adhesion of the *P. gingivalis* mutant strain deprived of PPAD was significantly lower than that observed for the wild-type strain (Karkowska-Kuleta et al., 2018). In addition, also the interaction with the host proteins may be altered since the citrullination of surface-exposed fungal proteins resulted also in the reduced binding of human plasminogen (Karkowska-Kuleta et al., 2020).

### Regulation of Hypha Formation and Biofilm Production

The master transcriptional regulatory network controlling *C. albicans* biofilm formation and filamentation includes proteins Bcr1, Tec1, Efg1, Ndt80, Rob1, and Brg1, while about a thousand target genes belong to this complex network (Nobile et al., 2012). In response to diverse environmental stimuli occurring in the host’s niche, various interrelated signaling cascades, including the cyclic adenosine monophosphate (cAMP)-dependent protein kinase A (PKA) pathway and the mitogen-activated protein kinase (MAPK) signal transduction pathways, are triggered to activate transcription factors controlling the change in fungal morphology (Basso et al., 2019). Influencing the expression of these regulatory genes during contact with bacteria may have far-reaching consequences for the candidal morphogenesis and existence in mixed biofilm. In the case of the interaction of fungi with streptococci, there is a wide variation in the observations of the effect on changing the fungal morphology following contact with different bacterial species, as described above. The studies of do Rosário Palma et al. (2019) showed that interactions of *C. albicans* standard strain ATCC 18804 with *S. mitis* during the formation of mixed biofilm in 24-well microtiter plates for 48 h, when streptococci were added to the fungal biofilm preformed for 2 h after initial adhesion of 10^7^/ml of *C. albicans* cells, resulted in the upregulation of genes involved in the biofilm formation and filamentation, like *BCR1* required for the formation of biofilm and regulation of genes encoding cell surface-associated proteins and *CPH1* involved in the filamentation (Nobile and Mitchell, 2005; Nobile et al., 2006; Maiti et al., 2015). Additionally, an increase with uncertain statistical significance was also detected for *EFG1*, the major transcriptional regulator involved in fungal morphogenesis and a key transcriptional activator of hypha-specific genes (HSGs) (Stoldt et al., 1997; do Rosário Palma et al., 2019). In contrary, in the same work, the downregulation of *BCR1* and *EFG1* was noticed under the same conditions for *C. albicans* ATCC 18804 interaction with *S. sanguinis* (do Rosário Palma et al., 2019). Moreover, the analyses of changes in the proteome of mature 48-h biofilm formed by *C. albicans* standard laboratory strain SC5314 after its 2-h exposure to 10^8^ heat-killed bacteria revealed the reduction in the expression of Efg1 protein upon the contact with *S. mitis* and *S. sanguinis*, as well as with *P. gingivalis*, *F. nucleatum*, and *A. actinomycetemcomitans* (Truong et al., 2020).

During the contact of *C. albicans* strain SC5314 with *S. oralis* at a ratio of 1:10, the *EFG1* gene was significantly upregulated mainly in the late stages of biofilm growth, resulting also in the increase in the gene expression of adhesin *ALS1* and stimulation of cross-kingdom mucosal biofilm formation (Xu et al., 2017), whereas under the contact of *C. albicans* strain SC5314 with *S. gordonii* for 1 h at 37°C, the upregulation of the *TEC1* gene involved in the regulation of filamentous growth was shown (Schweizer et al., 2000; Dutton et al., 2016a). In the work of Salvatori et al. (2020), the *C. albicans* floating microcolonies formed in the presence of heat-fixed culture supernatants from *S. gordonii* were characterized by the increase in the expression levels of genes *EFG1* and *HGC1*. These genes encode G1 cyclin-related protein specific for hyphae and crucial for hyphal morphogenesis (Zheng et al., 2004). In the studies of Chinnici et al. (2019), it was demonstrated that *C. albicans* knockout mutant strains deprived of transcription factors Sfl2, Brg1, Tec1, Tup1, Efg1, and Rim101 had reduced ability to form dual-species biofilms with *S. gordonii* as compared to wild-type bacterial–fungal biofilms, indicating positive regulation by these factors. On the contrary, in the studies performed by Montelongo-Jauregui et al. (2019), the employment of *C. albicans efg1Δ/Δ*, *brg1Δ/Δ*, and *bcr1Δ/Δ* mutant strains to the formation of mixed biofilms with *S. gordonii* in basal medium mucin artificial saliva showed no significant differences compared with the wild-type strains. Hence, the authors suggested the ability to restore biofilm formation of filamentation-defective *C. albicans* mutant strains by *S. gordonii* and no need for filamentation to interact with these bacteria. Such divergent observations invariably indicate the complexity of the mechanisms governing the interactions between streptococci and *Candida* and their significant dependence on growth conditions, the methodological approach applied, and environmental requirements.

One of the external factors influencing the morphology of fungi is the secreted quorum sensing molecules (QSM). Farnesol is the best-known QSM produced by *C. albicans*, acting as a diffusible filament-suppressing signal, and also inhibiting biofilm formation (Hornby et al., 2001; Ramage et al., 2002). Farnesol stops the transition from yeast-like cells to hyphae, mainly through an inhibitory effect on the Ras1-Cyr1/cAMP-PKA cascade (Polke et al., 2017; Wang et al., 2020). During the formation of mixed-species biofilm by *C. albicans* SC5314 and *S. gordonii*, the addition of farnesol to mixed biofilm culture did not inhibit hyphal formation as in monospecies biofilm. It could be an effect of the inactivation of fungal farnesol receptors by bacteria or influencing the fungal cAMP-PKA pathway or stimulation of another intracellular signaling pathway, which predominated the farnesol inhibitory signal; however, further detailed studies of the mechanisms are required (Bamford et al., 2009).

### Cell Wall Glucans, Mannans, and Chitin

*C. albicans* cell wall scaffold is composed of linear or branched polysaccharides including chitin and β-1,3-glucan, forming the inner layer of the wall, and β-1,6-glucan and mannan structures linked to cell wall proteins *via O-* and *N*-glycosidic bonds, which build the outer part of the cell wall being in immanent contact with the host and the environment (Hall and Gow, 2013; Klis et al., 2014). During the infection, these polysaccharides are recognized by different host receptors, including lectins or complement factors, and they strongly induce host defense mechanisms and innate immune response; however, they are also responsible for evading the human immune system and contribute to the spread of pathogens within an organism (Snarr et al., 2017).

The correct O-mannosylation of the surface-exposed fungal proteins provided by the activity of Mnn1 and Mnn2 proteins was indicated as necessary for the interactions of *C. albicans* with *S. gordonii* (Dutton et al., 2014). The formation of mixed biofilms by *C. albicans mnt1*Δ *mnt2*Δ mutant strains and bacteria was significantly disturbed, probably as a result of improper surface exposition of fungal cell wall adhesins (Dutton et al., 2014). On the contrary, in the studies performed by Montelongo-Jauregui et al. (2019), *C. albicans* mutant strains with deletions of genes encoding cell wall and biofilm matrix-related proteins, including Kre5 and Mnn9, did not exhibit major defects in the formation of dual species biofilms with *S. gordonii* in basal medium mucin synthetic saliva. Kre5 protein provides the appropriate amount and ratio of glucans in the cell wall and Mnn9 is responsible for proper cell wall proteins’ mannosylation (Southard et al., 1999; Herrero et al., 2004). Similar observations were demonstrated for *C. albicans* mutants devoid of transcription factors Rlm1 and Zap1 involved in the cell wall and biofilm matrix biogenesis (Nobile et al., 2009; Delgado-Silva et al., 2014; Montelongo-Jauregui et al., 2019). As other studies have also shown, the formation of mixed-species biofilm with *S. gordonii* resulted in downregulation of the *CHT2* gene, encoding GPI-linked chitinase necessary for normal filamentous growth and responsible for remodeling of chitin in the fungal cell wall (McCreath et al., 1995; Dutton et al., 2016a). These few reports suggest that mixed biofilm formation with streptococci may have some effect on fungal cell wall biogenesis and maintenance, but more comprehensive studies are still required.

When creating a mixed biofilm by *C. albicans* ATCC 10231 and *P. gingivalis* wild-type strain and mutant strain deprived of gingipains, the increase in the amount of protein on the cell surface was determined for Cht2 in normoxia, whereas in anoxia such a protein quantity enhancement was only observed for the mixed biofilm formed with the bacterial mutant strain (Bartnicka et al., 2019). Also, the amount of surface-exposed endo-1,3(4)-β-glucanase 1 (Eng1), the protein responsible for cell separation after budding, was higher in mixed biofilm formed under two investigated culture conditions, but only with the wild-type strain of bacteria, while for the Mp65 protein, responsible for the metabolism of cell wall glucan, the increase was observed under aerobic conditions after contact with mutant strain and under anaerobic conditions for both bacterial strains (Esteban et al., 2005; Sandini et al., 2007; Bartnicka et al., 2019). Moreover, changes were also noticed for protein Bgl2, a cell wall-associated 1,3-β-glucosyltransferase involved in cell wall remodeling, whose level increased in normoxia in biofilm formed with mutant strain, while for Phr1, cell surface glycosidase involved in glucan cross-linking also in normoxia, but for wild-type strain (Sarthy et al., 1997; Fonzi, 1999; Bartnicka et al., 2019). Moreover, some of these proteins were also indicated as susceptible for modifications by *P. gingivalis* PPAD, as the citrullination at most two places was identified with mass spectrometry analysis for Eng1, Bgl2, Phr1, and Mp65 (Karkowska-Kuleta et al., 2020). Importantly, in the case of the bacterial deiminase, the citrullination also requires a pre-hydrolysis of the protein by R gingipain to expose the C-terminal arginine (Goulas et al., 2015); however, the impact of these enzymatic modifications on the structure and activity of fungal enzymes still needs to be elucidated. Further investigations of these processes are required because the influence on the surface presence or activity of particular enzymes involved in the remodeling of *C. albicans* cell wall by accompanying bacteria might cause changes not only in its composition, structure, and rigidity but also in interactions with the host immune system, which may indirectly alter the pathogenic potential of fungi forming a mixed biofilm.

### Proteases and Other Hydrolytic Enzymes

*C. albicans* produce ten secreted aspartyl proteinases (Sap1-10), of which Sap1-8 are secreted to the extracellular milieu, while Sap9 and Sap10 are equipped with the GPI anchor and remain bound to the cell surface and act there for the rearrangement of molecules exposed by fungi on the cell wall (Aoki et al., 2011; Schild et al., 2011; Rapala-Kozik et al., 2018). Another major virulence factor with hydrolase activity secreted by *C. albicans* is also phospholipase D1 (Pld1) involved in the fungal invasion on host tissues (Dolan et al., 2004).

In the studies of Dutton et al. (2014), a consistent reduction in the abundance of Sap9 was found in proteomic analyses in result of the interaction between *C. albicans mnt1*Δ *mnt2*Δ mutant strain and *S. gordonii*. Therefore, it could be concluded that this protein may play an important role in modulating cross-kingdom interactions. In the continuation of this work, it was presented using the *C. albicans sap9*Δ mutant that this proteinase might contribute to the competition of *C. albicans* within oral microbial biofilms, as Sap9 may be involved in the degradation of salivary pellicle-binding sites for streptococci (Dutton et al., 2016b). When studying the biofilm formation process with the anaerobe *P. gingivalis*, after a 3-h contact of *C. albicans* ATCC 10231 and *P. gingivalis* wild strain in aerobic conditions, the level of *SAP9* gene expression was increased compared with monospecies fungal biofilm, whereas for *SAP3* and *SAP6* the gene expression did not change (Bartnicka et al., 2019). In contrast, when a bacterial gingipain-deficient strain ΔKΔRAB was used, only the level of *SAP6* expression was significantly increased. At anoxic conditions, the production of biofilm with this impaired strain resulted in the upregulation of *SAP3*, *SAP6*, and *SAP9*, while for wild-type strain only *SAP6* was upregulated under the conditions tested, without any changes for the genes of the other tested proteases (Bartnicka et al., 2019). The obtained results may provide some evidence for the role that proteinases may play during the formation of a mixed biofilm with *P. gingivalis*, but their specific functions still need to be elucidated. Additionally, at the level of protein production, the formation of mixed biofilm for 24 h in the simultaneous model of interaction in RPMI 1640 medium by *C. albicans* ATCC 10231 and *P. gingivalis* mutant strain at a ratio of 1:10 increased the amount of lysophospholipase 1 (Plb1), a lipolytic enzyme being an important candidal virulence factor (Leidich et al., 1998; Bartnicka et al., 2019).

In 2016, Cavalcanti et al. analyzed the relative changes in the expression of genes encoding proteinases Sap2, Sap4, Sap6, and phospholipase D1 (Pld1) during biofilm formation by *C. albicans* ATCC 90028, *S. sanguinis*, *S. mutans*, and *P. gingivalis* at anaerobic conditions for 72 h on the titanium surface. When biofilm was formed by fungi and *P. gingivalis*, the expression levels of *SAP2* and *SAP6* significantly increased, for *SAP4* it was reduced, and for *PLD1* it remained unchanged. In the presence of streptococci, the obtained results were comparable (Cavalcanti et al., 2016). The formation of mixed biofilm by *C. albicans* and *S. sanguinis*, *S. gordonii*, *A. odontolyticus*, and *A. viscosus* cultured in 5% CO_2_/95% air for 72 h resulted in an increase in the gene expression for proteinases *SAP4* and *SAP6* and phospholipase D1 (*PLD1*) compared with single-species fungal biofilm. The additional presence of *P. gingivalis* in this complex did not induce changes in high expression levels of *SAP4* compared with monospecies biofilm, but it resulted in a decrease in *SAP6* expression when compared to not only monospecies biofilm but also fungal–bacterial biofilm without *P. gingivalis*, while the expression of *PLD1* decreased in the biofilm containing *P. gingivalis* compared with the mixed biofilm formed without this anaerobic bacterium but did not change compared to the single species fungal biofilm (Morse et al., 2019). These observations may indicate that a specific composition of bacterial species possesses the capability to modulate interactions within the complex microbial community.

## Influence of Periodontal Biofilm Formation on Diagnosis and Treatment

Persistent infections located within the gingival pockets might be the source of pathogens capable to spread further in the host organism, causing disseminated infections at distant locations or contributing to the development of systemic health-threatening diseases like atherosclerotic disease, rheumatoid arthritis (RA), and respiratory or gastrointestinal illnesses, especially in the cases of immunosuppression or other predisposing factors (Paju and Scannapieco, 2007; Han and Wang, 2013; Vieira Colombo et al., 2016). The correct diagnosis and effective treatment of such subsequent microbial infections are not always quickly feasible and trouble-free. Likewise, it is difficult to prevent effectively the influence of a microbial factor on the development or course of systemic secondary diseases in humans. In the case of invasive candidiasis, including deep-seated infections of inner organs and candidemia, the standard diagnostic method used is to cultivate the fungi from tissue samples or blood. Nevertheless, its main disadvantages are the possibility of detection of only viable and culturable *Candida* cells, prolonged waiting time for results, and low sensitivity, as almost half of the cases of invasive candidiasis remain undiagnosed (Clancy and Nguyen, 2013; Clancy and Nguyen, 2018). Other currently proposed diagnostic tests, in addition to the PCR and T2 Candida nanodiagnostic panel, are based on the detection of *Candida* antigens or, more often, of antibodies against different molecules exposed by the pathogen, including the components of the fungal cell wall, such as mannan and β-1,3-glucan, and antibodies against antigens located on the cell wall of *C. albicans* hyphal forms, i.e., CAGTA—*Candida albicans* germ tube antibodies (Mikulska et al., 2010; Avni et al., 2011; Martínez-Jiménez et al., 2014; Mylonakis et al., 2015; León et al., 2016). These tests presuppose the development of an immune response by the host during such systemic candidiasis and the production of specific antibodies directed against particular surface-exposed fungal antigens (Bouza et al., 2020). The antigen-directed tests are frequently used in combination with tests detecting antibodies to increase diagnostic sensitivity (Ahamefula Osibe et al., 2020).

**Figure 3:**
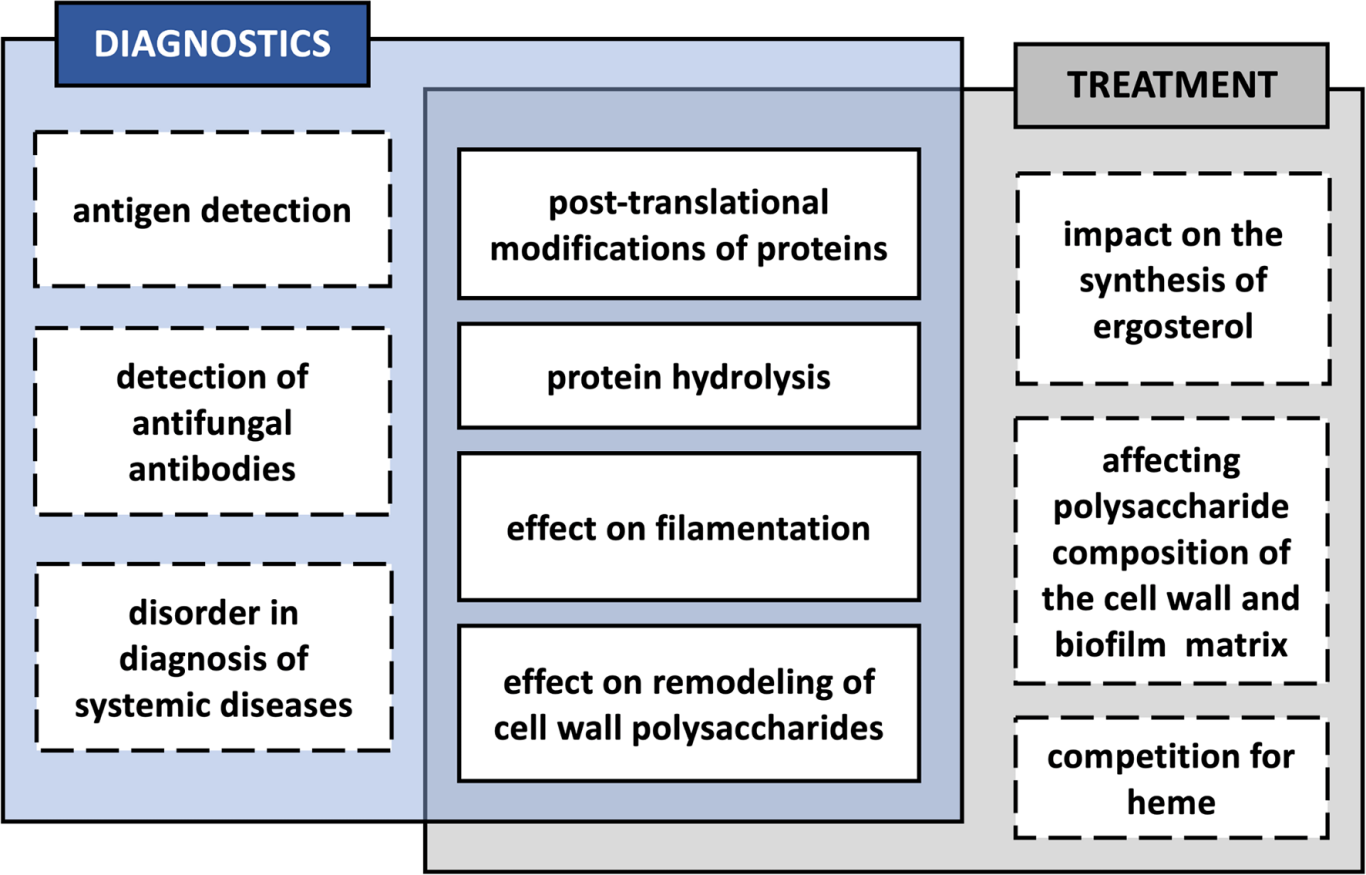
Potential effects of the interaction of *Candida albicans* with bacteria in a biofilm, influencing the diagnosis and treatment of mixed infection.

There is always a possibility that modifications of microbial molecules taking place during the coexistence of many different microorganisms in a biofilm during severe periodontitis may also affect the course of the subsequent spread of pathogens, and thus also the correct diagnosis of such infections and an effective method of their treatment (**Figure 3**). One such posttranslational protein modification in the case of *C. albicans* is citrullination of surface-exposed proteins in the reaction catalyzed by *P. gingivalis* PPAD, as the change in the net charge of a protein may affect its structure and function, also within specific protein domains, and also its immunomodulatory properties, meaning the ability of antigen presentation and recognition by the immune system (Doyle and Mamula, 2012). The modification of arginine to citrulline might result in the altered presentation of the modified peptides to CD4^+^ T cells, as these two amino acids differ in the affinity to the binding pockets of HLA-DR (human leukocyte antigen–DR isotype) proteins in favor of the latter (Scally et al., 2013). Together with certain genetic predispositions, the citrullination of human proteins may therefore be associated with the development of autoimmune diseases and the production of anti-citrullinated protein antibodies (ACPAs), e.g., in the course of RA, and one well-known example of such modified proteins associated with self-intolerance in RA is human α-enolase (Schellekens et al., 1998; James et al., 2014; Gerstner et al., 2020). As indicated by Lundberg et al. (2008), *P. gingivalis* infection localized in the periodontium, resulting in the citrullination of bacterial enolase, might be related to cross-reactivity of the antibodies specific to an immunodominant epitope of human citrullinated α-enolase. The question remains whether the citrullination of the fungal enolase that occurs during mixed *C. albicans* infection with *P. gingivalis* may also exert such effects implying the potential commencement of autoimmunity. It was previously demonstrated that PPAD might also citrullinate human α-enolase within the sequence that is responsible for the disease-specific antibody response in patients with RA (Lundberg et al., 2008; Wegner et al., 2010). Therefore, this assumption is quite reasonable, as in fungal enolase, the citrullination *via* bacterial deiminase targets the R_333_ residue located within a sequence homologous to that in human enolase, which also contains a modified arginine residue being identified as reactive with sera of RA patients (Lundberg et al., 2008; Karkowska-Kuleta et al., 2020).

As mentioned above, except *C. albicans* enolase, also other fungal surface-exposed or secreted proteins might be citrullinated by PPAD (Karkowska-Kuleta et al., 2018; Karkowska-Kuleta et al., 2020). Some of these proteins, including Tdh3, Pgk1, Pdc11, Bgl2, Mp65, Pga4, Pra1, Ssb1, and Ssa2, were indicated in other studies as immunoreactive during systemic fungal infections, and they are currently considered as potential diagnostic markers (Pitarch et al., 1999; Pitarch et al., 2004; López-Ribot et al., 2004; Mochon et al., 2010; Pitarch et al., 2016). In the studies of Vaz et al. (2021), several proteins secreted by *C. albicans* hyphae were postulated as immunoreactive in patients with catheter-associated and non-catheter-associated invasive candidiasis. Of these proteins, *C. albicans* Eno1 and Bgl2 showed antibody-reactivity patterns allowing the classification of patients with invasive candidiasis, whereas the antibody response observed for Tdh3 was distinctive for catheter-associated invasive candidial infection. Cell wall protein Mp65 was described as the main target of human T-cell response to *C. albicans* and has also been considered as a new objective in the diagnosis of candidemia (Gomez et al., 1996; Berzaghi et al., 2009; Torosantucci et al., 2017). Furthermore, the elevated levels of anti-Bgl2p antibodies and the seropositivity of antibodies against Pgk1 were demonstrated as independent predictors of systemic candidiasis by Pitarch et al. (2006). The modification of these proteins by bacterial PPAD during coinfection may likely affect their serodiagnostic usefulness, but further research on this issue is certainly required. Importantly, in the case of bacterial deiminase, the structure of the active center of the enzyme determines its predominant affinity for the C-terminal arginine, and not for arginine located within the protein chain, as is the case with human deiminases (Goulas et al., 2015). This implies the need for peptide bond hydrolysis by proteases after the C-terminal arginine residue, and this activity is attributed to arginine gingipains A and B (RgpA and RgpB) secreted and surface-exposed by *P. gingivalis* (Potempa et al., 2003). Fungal proteins may be degraded to a varying degree by bacterial proteases depending on their structure and the conditions of the microenvironment (Bartnicka et al., 2019). Therefore, a further question of how proteolytic processing of these proteins might alter the host immune response to fungal antigens remains to be explored.

Furthermore, in the case of direct interactions between *F. nucleatum* and *C. albicans* resulting in the inhibition of fungal filamentation (Bor et al., 2016), the diagnosis of fungal infection based on the detection of host antibodies directed against antigens presented on the surface of germ tubes (CAGTA) may be significantly hampered. A similar observation was reported for *C. albicans* in contact with *P. nigrescens* (Thein et al., 2006). Hence, similar conclusions could be drawn about the consequences for diagnostics. Also, the arrival of a new member in the consortium of *C. albicans* and *S. sanguinis*, *S. gordonii*, *A. odontolyticus*, and *A. viscosus*, which stimulated the formation of hyphae by fungi, may partially inhibit the filamentation process as shown for *P. gingivalis* introduced to this complex biofilm and thus make detection of *C. albicans* hyphae more difficult. Nevertheless, under the tested conditions, in the mixed biofilm composed of these species, the level of filamentation was still higher than in a single fungal biofilm, but with uncertain statistical significance (Morse et al., 2019). Also, the varied influence of streptococci on fungal filamentation may have an impact on diagnostics based on the detection of hyphae.

Currently, a promising approach for the diagnosis of periodontal disease is the analysis of the metabolome of fluids collected from the oral cavity, including saliva or gingival crevicular fluid (Mikkonen et al., 2016). They contain numerous different molecules being the result of metabolic processes taking place at the site of infection, including both those derived from the host and those produced by inhabiting microorganisms. Any change in the delicate balance between the host and its microbiome, or the appearance of a new component in the latter, may be reflected in the subtle differentiation in the set of metabolites found in the niche analyzed (Na et al., 2021; Thomas et al., 2021). Possibly, the analysis of changes in the amount of certain metabolites could give information about the rate and direction of the development of periodontal disease and enable monitoring the treatment process; however, it still requires further extensive research, given the significant impact of systemic diseases on the composition of this fluid and the fact that results of studies on different patient groups are often contradictory (Nguyen et al., 2020; Baima et al., 2021).

One of the proposed biomarkers of periodontal disease is prostaglandin E_2_ (PGE_2_), whose production by fibroblasts and smooth muscle cells increased under inflammatory conditions (Båge et al., 2011; Elabdeen et al., 2013). As it has been demonstrated by Erb-Downward and Noverr (2007), *C. albicans* may also synthesize PGE_2_, but *via* distinct pathways than in human cells, using fatty acid desaturase homolog Ole2 and a multicopper oxidase homolog Fet3. Therefore, fungal contribution to mixed biofilm during aggressive periodontal disease could disturb the analysis of the level of PGE_2_ in gingival crevicular fluid and influence disease diagnosis. In addition, it has been shown that PGE_2_ stimulates the production of germ tubes by *C. albicans*, which may favor the detection of fungi based on filamentation-related antigens (Noverr and Huffnagle, 2004).

The oral microbiome dysbiosis during periodontal disease might result also in changes in salivary metabolomics, including repeatedly indicated upregulation in salivary levels of valine, isoleucine, phenylalanine, tyrosine, proline, succinate, butyrate, and cadaverine (Nguyen et al., 2020; Baima et al., 2021). In *C. albicans*, the increased polyamine levels control the change from yeast-like cells to filamentous forms (Herrero et al., 1999) and their synthesis is related to the activity of polyamine biosynthetic enzymes, such as ornithine decarboxylase and spermidine synthase (Hamana et al., 1989). Therefore, the observed changes in the level of polyamines may not only depend on the presence of *Candida* yeasts in the microbiome in periodontal tissues but also affect the morphology of the fungi and their detection based on the presence of hyphae.

Moreover, variations in the levels of lactate, pyruvate, N-acetyl groups, and methanol in saliva might also be predictive for oral health or disease (Baima et al., 2021). Upregulation has been observed in healthy subjects for the latter two, and reports of changes in lactate and pyruvate levels are contradictory depending on the type of studies (Baima et al., 2021). The differentiation in access to carbon sources significantly influences the structure of *C. albicans* cell wall, as well as its proteome and secretome (Ene et al., 2012a). Lactate-grown cells exhibit higher levels of proteins involved in β-glucan remodeling, including glucanosyltransferases Pga4, Phr1, and Phr2 and exo-glucanase Xog1, whereas for glucose-grown cells other cell wall-organizing enzymes prevailed, like Bgl2 (Ene et al., 2012b). Such fluctuations in the architecture of the cell wall related to the availability of different carbon sources may also be important in the diagnosis based on the presence and exposition of different polysaccharides of the candidial cell wall or secreted proteins.

In addition to diagnostics, also the prevention of infections caused by *Candida* is particularly important. Currently, two *Candida* vaccines are undergoing clinical trials and the preliminary results are encouraging (Oliveira et al., 2021). The first type of vaccine, PEV7, is based on the recombinant *C. albicans* aspartyl proteinase 2 (Sap2), and the second type, NDV-3/NDV-3A, on the recombinant N-terminal part of *C. albicans* agglutinin-like sequence protein (Als3) (De Bernardis et al., 2012; Schmidt et al., 2012; Edwards et al., 2018). However, in the case when the epitopes recognized by the antibodies produced during the protective response will be altered due to the intermicrobial interaction-dependent modifications, the effectiveness of the immunization may adversely differ from that assumed. One such example might be protein Als3, demonstrated as susceptible for citrullination within the N-terminal domain, where two citrulline residues at positions 175 and 188 after incubation with *P. gingivalis* PPAD were identified (Karkowska-Kuleta et al., 2020).

As with diagnostics, the coexistence of *C. albicans* with bacteria in polymicrobial biofilms may also have an impact on the treatment of mixed infections. The composition of membrane sterols is a key factor of azole resistance at the intermediate and mature stages of biofilm development (Mukherjee et al., 2003). Therefore, the alternation in sterol synthesis or their membrane incorporation caused by the presence of bacteria in the mixed community might be an important contribution to the antifungal resistance or susceptibility by *C. albicans* (Bhattacharya et al., 2020). In *C. albicans* biofilms treated for 2 h with heat-killed bacteria *S. mitis*, *S. sanguinis*, *P. gingivalis*, *F. nucleatum*, or *A. actinomycetemcomitans*, the expression levels of proteins Erg11—lanosterol 14-alpha-demethylase, a key enzyme in ergosterol biosynthesis, and Erg13—3-hydroxy-3-methylglutaryl coenzyme A synthase involved in sterol biosynthesis, were somewhat reduced (Kirsch et al., 1988; Liu et al., 2005; Truong et al., 2020). However, further studies in the context of fungal resistance to azoles were not conducted in that study (Truong et al., 2020). Also, the experimental design concerns heat-killed bacteria, a procedure that disrupts the native surface disposition; therefore, a verification of these results during biofilm formation could shed new light on the influence of these bacteria on sterol synthesis. Furthermore, it was previously demonstrated that upregulation of *ERG11* gene expression in response to fluconazole was detected after about 2 h since stimulation with an antifungal drug; therefore, investigating these relations at extended time intervals should be considered (Henry et al., 2000).

In the studies of the formation of mixed biofilms by *S. gordonii* and *C. albicans* in basal medium mucin synthetic saliva, the increased resistance of dual-species biofilms compared with single-species biofilms to a combinatorial therapy consisting of clindamycin and either fluconazole, amphotericin B, or caspofungin was demonstrated (Montelongo-Jauregui et al., 2016). These findings were further confirmed with the analysis of the formation of dual-species biofilms between these two species on the surface of titanium discs, where the increased resistance to combinations of clindamycin and the abovementioned antifungal drugs used at high concentrations was demonstrated again (Montelongo-Jauregui et al., 2018). A continuation of these studies allowed to demonstrate that during dual-species biofilm formation, *C. albicans* adhesin (*als3*Δ/Δ) and filamentation deletion mutant strains *bcr1*Δ/Δ, *efg1*Δ/Δ, and *brg1Δ/Δ* displayed the resistance to antimicrobial treatment with amphotericin B and clindamycin similar to those formed by their respective wild type strains (Montelongo-Jauregui et al., 2019). However, in the case of mixed biofilms formed by *S. gordonii* and *C. albicans kre5Δ/Δ* and *mnn9Δ/Δ* mutant strains, the increased bacterial susceptibility to clindamycin was observed, indicating the protective role of fungal biofilm matrix glucans and mannans against antibiotics (Montelongo-Jauregui et al., 2019). Similar conclusions concerning the bacterial protection against antimicrobials by *C. albicans* were drawn for the streptococcal resistance to ampicillin and erythromycin in cross-kingdom biofilms, albeit without indicating the exact mechanism (Chinnici et al., 2019).

It could be also assumed that while *C. albicans* formed a biofilm with *P. gingivalis* and there were changes observed in the frequency of surface exposition of enzymes related to remodeling of the fungal cell wall, as well as their modifications by bacterial enzymes were identified, this could have an impact on the composition of the cell wall polysaccharides and the biofilm matrix. An indirect effect of such changes would be the variability of the mixed biofilm in resistance to the antibacterial or antifungal drugs used; nevertheless, this problem requires further detailed investigations. In the studies by Taff et al. (2012), it was demonstrated that *C. albicans* cell wall modifying enzymes Bgl2, Phr1, and Xog1 are involved in β-1,3-glucan transport and its accumulation in the biofilm matrix and their activity is related to the resistance of fungal cells growing in the biofilm to the treatment with fluconazole. The mechanism of this phenomenon is related to the drug sequestration by the biofilm matrix and preventing reaching the target cells (Taff et al., 2012). As two of these enzymes—Phr1 and Bgl2—are present on the cell surface in an increased amount in the mixed biofilm formed with *P. gingivalis* and are also susceptible to the modifications by bacterial enzymes (Bartnicka et al., 2019; Karkowska-Kuleta et al., 2020), it could be hypothesized that in the case of such dual-species biofilm it could have an impact on its resistance to the antifungal drug, albeit this issue still requires further research. On the other hand, studies of mixed biofilms also showed that under conditions of reduced heme availability within the biofilm formed by *C. albicans* and *P. gingivalis*, the competition for heme augments the virulence of *P. gingivalis*, which was also reflected in the increase in bacterial resistance to cefazolin and sulfamethoxazole tested with the disk diffusion method (Guo et al., 2020).

Recently, the studies conducted by Young et al. (2020) concerning the role of *C. albicans* as a keystone commensal in polymicrobial oral biofilms associated with periodontitis/denture stomatitis showed that the presence of fungi in such biofilm did not affect their susceptibility to short-term used biofilm eradication agents. Such biofilms were formed in the presence or absence of *C. albicans* by *S. oralis*, *S. mitis*, *S. intermedius*, *F. nucleatum*, *F. nucleatum ssp vincentii*, *Actinomyces naeslundii*, *Veillonella dispar*, *P. gingivalis*, *P. intermedia*, and *A. actinomycetemcomitans* and then analyzed for biofilm thickness and metabolic activity, as well as for bacterial and fungal load following 10-min treatment with chlorhexidine gluconate, EDTA, potassium iodide, or antifungal drug miconazole (Young et al., 2020). These studies showed that under the conditions applied, the presence of *C. albicans* in multispecies biofilm did not provide significant protection for the microbiota against the range of treatment agents used, compared with bacterial biofilms formed without fungi. Moreover, the attention was drawn to the indispensable necessity to mechanically remove such biofilms during treatment as an effective introduction to further chemical therapy (Young et al., 2020). The data published so far show that the analysis of the influence of bacterial–fungal biofilm formation on their resistance to the applied treatment is particularly complex and still requires extensive examination.

Further comprehensive studies on the interrelationship between bacteria and fungi in mixed biofilm in the course of periodontitis may in the future help in designing more precise and effective methods of prevention and diagnosis of secondary diseases, as well as in combating the resistance of such biofilms to the applied treatment.

## Author Contributions

DS, JK-K, and MR-K contributed to the conception and design of the study. MG-G, MSo, MSu, KK, EW, and MZ performed the data searching and wrote sections of the manuscript. DS, JK-K, and MR-K wrote the first draft of the manuscript. AK performed the final corrections. All authors contributed to manuscript revision and read and approved the submitted version.

## Funding

This work was financially supported by the National Science Centre of Poland (grant no. 2019/33/B/NZ6/02284 awarded to MR-K).

## Conflict of Interest

The authors declare that the research was conducted in the absence of any commercial or financial relationships that could be construed as a potential conflict of interest.

## Publisher’s Note

All claims expressed in this article are solely those of the authors and do not necessarily represent those of their affiliated organizations, or those of the publisher, the editors and the reviewers. Any product that may be evaluated in this article, or claim that may be made by its manufacturer, is not guaranteed or endorsed by the publisher.
